# Ataxia Telangiectasia Arising as Immunodeficiency: The Intriguing Differential Diagnosis

**DOI:** 10.3390/jcm12186041

**Published:** 2023-09-19

**Authors:** Federica Cavone, Susanna Cappelli, Alice Bonuccelli, Sofia D’Elios, Giorgio Costagliola, Diego Peroni, Alessandro Orsini, Rita Consolini

**Affiliations:** 1 Pediatrics Unit, Department of Clinical and Experimental Medicine, University of Pisa, 56126 Pisa, Italy; f.cavone@studenti.unipi.it (F.C.); giorgio.costagliola@hotmail.com (G.C.); diego.peroni@unipi.it (D.P.); 2 Section of Clinical and Laboratory Immunology, Division of Pediatrics, Department of Clinical and Experimental Medicine, University of Pisa, 56126 Pisa, Italy; su.cappelli@ao-pisa.toscana.it (S.C.); s.delios@ao-pisa.toscana.it (S.D.); 3 Section of Pediatric Neurology, Division of Pediatrics, Department of Clinical and Experimental Medicine, University of Pisa, 56126 Pisa, Italy; al.bonuccelli@ao-pisa.toscana.it (A.B.); aorsini.md@gmail.com (A.O.)

**Keywords:** ataxia, immunodeficiency, inborn errors of immunity, movement disorders

## Abstract

Ataxia telangiectasia (AT) is a rare disease characterized by the early onset and slow progression of neurodegenerative defects, mainly affecting the cerebellum, associated with immunodeficiency and teleangiectasias. Ataxia is the hallmark of the disease and usually its first manifestation. Overt cerebellar ataxia usually becomes evident between 16 and 18 months of age, after the onset of walking, and is characterized by frequent falls and an ataxic gait with an enlarged base. We report the case of a child who first presented with serious recurrent infectious, without exhibiting neurological symptoms. The patient was initially diagnosed with combined immunodeficiency (CID) of unknown etiology for nearly 3 years, before he was definitively diagnosed with ataxia telangiectasia.

## 1. Introduction

Ataxia telangiectasia (AT) is a multisystemic autosomal recessive inherited disease associated with defective DNA repair mechanisms and clinically characterized by cerebellar ataxia, oculo-cutaneous telangiectasias, immunodeficiency, radiosensitivity, and high cancer susceptibility. In adulthood, the key signs of the disease are usually overall expressed, but the onset time of the different manifestations and the rapidity of progression of the disease lead to phenotypic variability in its presentation in childhood. Mild trunk ataxia with anteroposterior and lateral oscillations and mild to moderate trunk hypotonia with hypotonic posture may represent early clinical signs, appearing after the sixth month of age. However, the majority of children appear normal at birth and their psychomotor development is not different from that of healthy peers until the time of acquisition of autonomous walking. The dyssynergy between the trunk and limbs makes their walking uncertain with continuous adaptation of the support base. Moreover, cerebellar ataxia is usually the first manifestation of the disease. We report the case of a child who firstly presented with serious recurrent infections and had been diagnosed with combined immunodeficiency (CID) of unknown etiology for nearly 3 years before he was definitively diagnosed with AT. As AT diagnosis is particularly challenging in cases arising only with the features of immunodeficiency, we focus on the differential diagnostic approach of patients presenting with immunodeficiency and neurological defects.

## 2. Case Report

The patient was a male who came to our observation at the age of 3 years and 8 months. He had previously received a diagnosis of CID in an another immunological center. His medical history reported recurrent upper and lower airway infections since one year of age, requiring intravenous (IV) antibiotic treatment. Immunologic examinations revealed low expression of T lymphocyte receptor excision circles/DNA and kappa-deleting recombination excision circles (TREC and KREC) on neonatal spot, low IgA (<−2 DS) and IgG (<−2 DS), elevated IgM (1450 mg/dL), lymphopenia (2198/mmc), low T cell subpopulations (CD4+: 16%, 391/mmc; CD8+: 13%, 312/mmc), reduced CD4 naive T cells (4.8%), and reduced lymphocyte proliferation evaluated with carboxyfluorescein succinimidyl ester (CFSE) flow cytometry. He underwent molecular analysis of IL2Rγ and the intron/exon junctions, CD40, and CD40L, which were found to be negative. The CGH array was negative. Replacement therapy with immunoglobulins (first intravenous and then subcutaneous) and prophylactic therapy with sulfamethoxazole-trimethoprim against *Pneumocystis jiroveci* were introduced. At the age of three years and six months, chest computed tomography (CT) was performed, showing bilateral bronchiectasis of cylindrical type, bronchiolectasis, and bilateral interstitial nodular thickenings associated with acinar opacities. After refusing to undergo hematopoietic stem cell transplantation (HSCT), the family turned to our center.

**Figure 1:**
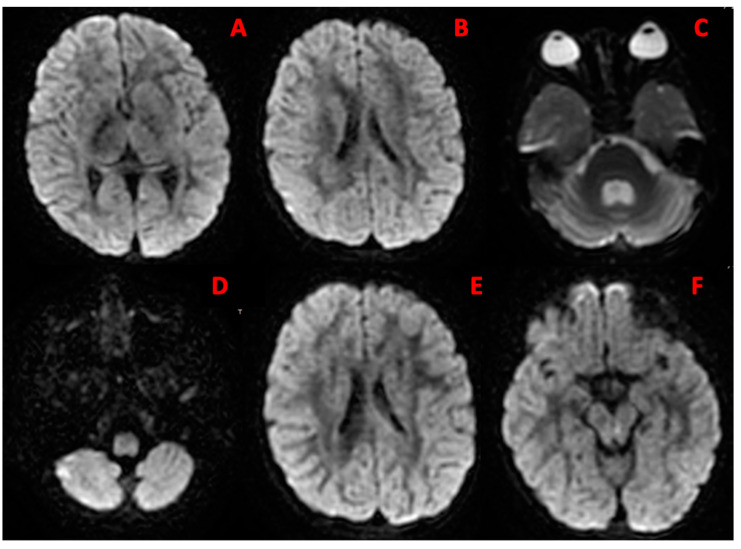
Brain MRI features. Brain MRI features: (**A**,**F**) gray matter reduction; (**B**,**E**) enlargement of the lateral ventricles; (**C**,**D**) cerebellar atrophy.

At our physical examination, the child was in fair general condition; pulmonary auscultation demonstrated crackles on both lower lung bases. The neurological examination was overall negative. Notably, mild postural uncertainty of the trunk with slight oscillations was observed. The previous therapy was confirmed and antibiotic prophylaxis with azithromycin was added, due to the recent history of numerous bacterial infections. After one year, at the age of 4 years, the child had developed proximal hypotonia and progressive postural instability of both the trunk and head, with worsening swaying. He showed sudden uncoordinated and late postural trunk adjustments. Romberg’s test was positive. Bradykinesia was evident in some movements, particularly of the upper limbs. Walking appeared uncertain due to a level of dyssynergy between the trunk and limbs, already visible in upright posture. Brain and cervical spine magnetic resonance imaging (MRI) showed expansion of the IV ventricle and the liquor spaces surrounding the cerebellum, as a consequence of cortical–subcortical cerebellar atrophy (Figure 1).

**Figure 2:**
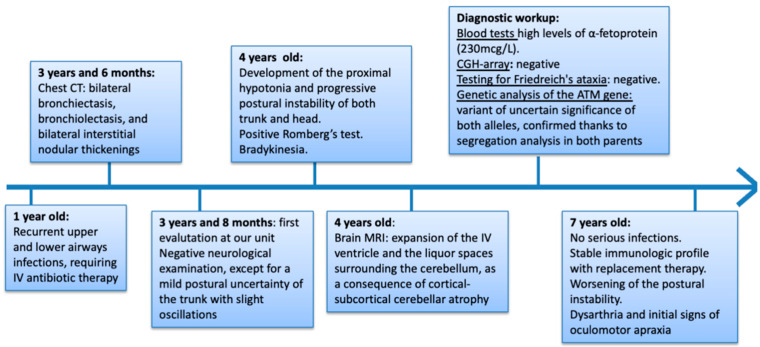
Timeline from disease onset to definitive diagnosis.

In light of the MRI, blood tests were performed, revealing high levels of α-fetoprotein (AFP: 230 mcg/L). CGH array did not reveal any microduplication or microdeletion, and DNA tests for the expansion of repeated triplets in the genes associated with Friedreich’s ataxia (FXN) and with other forms of ataxia (ATXN1, ATXN2, ATXN3, CACNA1A, ATXN7, ATXN8OS, PPP2R2B, TBP, ATN1) were also negative. Genetic analysis of the ATM gene showed a variant of uncertain significance of both alleles (p.V1268 *- p.A1299fs * 3). Therefore, genetic counselling was performed and segregation analysis revealed a heterozygous mutation of the ATM gene in both parents. Based on the clinical phenotype (cerebellar ataxia, recurrent lower airway infections), immunological profile (humoral and cellular deficiency), elevation of AFP, and biallelic mutations of the ATM gene, the diagnosis of ataxia telangiectasia was established (Figure 2).

At the age of seven, the patient did not report serious infections. His immunologic profile was stable; replacement therapy and prophylaxis against opportunistic infections were continued. At the neurological examination, the postural instability had worsened, with difficulty in maintaining both standing and sitting positions. Walking was discontinuous with a mowing appearance. The child displayed dysarthria, with slow and slurred speech, initial signs of oculomotor apraxia, and initial conjunctival telangiectasias.

## 3. Discussion

Ataxia telangiectasia is an autosomal recessive disorder characterized by progressive cerebellar degeneration, immunodeficiency, radiosensitivity, and cancer susceptibility. The prevalence of the disease is estimated to be less than 1–9:100,000 individuals, and the incidence ranges from 1:40,000 to 1:300,000 [1]. AT is caused by a recessive biallelic mutation in the ataxia-telangiectasia-mutated (ATM) gene [2].

This gene, located on chromosome 11q22-23, encodes a protein kinase involved in the repair of errors during DNA duplication and in the control of the cell cycle [1]. The absence of ATM protein prevents DNA double-strand breaks (DSBs) from being repaired by mediating early cellular responses to DSBs, generated during metabolic processes or by DNA-damaging agents [2]. It also activates cell cycle checkpoints and induces apoptosis in response to DSBs [2,3]. The role of ATM-mediated checkpoints in blocking the long-term persistence and transmission of un-repaired DSBs in developing lymphocytes has been highlighted [3]. Moreover, its mutation causes genomic instability, which implies radiosensitivity, neurodegeneration, immunodeficiency, and cancer susceptibility [4]. Ataxia is the main neurological sign of the disease and is usually the first clinical manifestation that occurs in childhood. The first symptoms in the form of truncal ataxia are seen usually in the first year of life as excessive instability of the truncus and of the head in sitting and standing posture, mild to moderate proximal hypotonia, and executive slowness in the upper limbs and the manipulation of objects. The onset of walking can be characterized by an ataxic gait with an enlarged base. Ataxia is progressive; at the beginning of the second decade of age, most children must rely on a wheelchair. During the disease course, involuntary movements such as chorea, athetosis, dystonia, and myoclonia [1] and progressive speaking difficulties may appear. At school, patients have difficulties in reading, due to a lack of coordination of eye movements, and in the finer motor functions such as writing or the use of utensils for eating [1,4]. With advancing age, patients manifest weakness, muscular atrophy, sensory deficit, absent deep reflexes, and peripheral neuropathy [5].

The other AT cardinal neurologic sign is oculomotor apraxia, with variable onset but present in almost all patients. MRI demonstrates the diffuse and progressive degeneration or atrophy of the cerebellar vermis and hemispheres [6]. Positron emission tomography (PET) imaging shows a reduction in glucose metabolism in the cerebellum and its increase in the globus pallidus, underlying the reduction in motor performance [1]. Telangiectasias on the bulbar conjunctiva is the second pathognomonic sign of the disease, which generally appears after the onset of ataxia, at 5–8 years of age [7], without impairing vision [1]. It can also occur in the sun-exposed areas of the skin, such as the face and ears. Cardiovascular defects, growth retardation, multiple endocrine dysfunction, premature aging, and insulin resistance are often included in the complex clinical picture of AT patients [1,4].

Immunodeficiency is present in approximately 60% of patients, involving both cellular and humoral immunity with variable degrees of severity. The most common abnormalities are low levels of one or more isotypes of immunoglobulins, failure to generate a specific antibody response to vaccines, and lymphopenia, especially affecting T lymphocytes. A small percentage of AT patients may have elevated levels of IgM in combination with IgG and/or IgA deficiency. IgE levels are also reduced. The anomalies of cellular immunity are heterogeneous, including a reduction in the cell subsets CD3+, CD4+, and CD45+ cells, whereas CD8+ cells are usually normal or slightly increased [8]. The T cell repertoire is limited, and the mitogen response and cytokine production are poor. For most patients, the pattern of immunodeficiency observed in early life persists throughout the lifetime; therefore, immunological analyses must be monitored unless the individual develops severe infections [9], as these remain the primary cause of death in AT patients despite early and aggressive treatment. Interestingly, a recent paper indicates the feasibility of using the TREC/KREC assay for early AT detection [10]. Immunodeficiency underlies a clinical picture characterized by recurrent, mainly sino-pulmonary, infections that become chronic in one third of patients, leading to bronchiectasis, pulmonary fibrosis, and interstitial lung disease (ILD). Interestingly, an association between elevated serum IL-6 and IL-8 levels and a reduction in lung function has been found in AT, suggesting that the evaluation of these parameters allows one to identify patients who are more susceptible to developing chronic lung disease [11].

Additionally, cutaneous granulomas are a recognized phenomenon in AT, with extra-dermal manifestations rarely found in bone and synovial fluid [12].

AT patients are more susceptible to ionizing radiation and have a higher incidence of cancer (approximately 25% lifetime risk). Lymphomas and leukemia often occur in people aged under 20 years, while adults are susceptible to both lymphoid and solid tumors including breast, liver, gastric, and esophageal cancers. It has been reported that residual ATM kinase activity suggests a protective effect against the development of cancer in AT childhood [13].

The current treatment modalities for AT involve symptomatic and supportive care and require a multidisciplinary approach for both pharmacological and non-pharmacological interventions [14]. Neurological manifestations progress and compel the patients to use a wheelchair at the beginning of the first decade. In some cases, a modest improvement can be obtained with the use of L-Dopa, dopamine agonists, and, occasionally, anticholinergics [15]. A short-term improvement in ataxia can be obtained using steroids [16]. Short-term beneficial effects on neurological symptoms (as evidenced by the assessment of neurological impairment rating scales) with the use of betamethasone have been reported [17]. To avoid the adverse long-term effects of steroid administration, an innovative method of delivering the drug, by incorporating dexamethasone within autologous erythrocytes, was developed [17].

The knowledge that chronic inflammation and immune activation mediate the neurodegenerative process and the multisystemic damage of AT patients has suggested promising experimental and clinical therapeutic strategies. This is based on the hypothesis that pharmacologically targeting proinflammatory markers can contribute to preventing the progression of inflammation and cancer [16]. Furthermore, timely AT diagnosis will allow the prevention of the development of severe infections and improve quality of life, as well as commencing genetic counselling of the family and implementing cancer prevention measures [18].

### The Differential Diagnosis

Ataxia telangiectasia is a very rare disease; the differential diagnosis includes essentially three disorders: cerebral palsy, congenital ocular motor apraxia, and Friedreich’s ataxia. Cerebral palsy is caused by the malformation or early damage of cerebellar structures and describes a non-progressive disorder of motor function, resulting in ataxic palsy in approximately 5–10% of all cases of cerebral palsy. These patients present with a decrease in muscle tone and experience difficulties in movement coordination, being the intention tremor the most common manifestation [19]. Congenital ocular motor apraxia (COMA) is a rare delayed developmental disorder of the visual saccades. COMA arises early and improves over time, whereas, in AT patients, similar saccadic difficulties worsen typically in early school age [20]. Friedreich’s ataxia (FRDA) is the most prevalent autosomal recessive cerebellar ataxia and the most common genetic cause of ataxia in children, starting typically between 10 and 15 years of age. Unlike FRDA, AT patients have telangiectasias, oculomotor apraxia, early absence of tendon reflexes, and an elevated level of AFP [21]. Since, in the presenting case, AT arose with a clinical picture compatible with an inborn error, leading to an initial diagnosis of combined immunodeficiency of unknown etiology, we focused on primary immune defects whose clinical phenotype included neurologic manifestations, to contribute to the improvement of the differential diagnosis process. The definition of several inborn errors of immunity—ataxia-telangiectasia-like disorder (ATLD), adenosine deaminase (ADA) deficiency, purine nucleoside phosphorylase (PNP) deficiency, Nijemegen breakage syndrome (NBS), DNA ligase IV deficiency (LIG4), Cernumnos/XLF deficiency, common variable immunodeficiency disease (CVID), immunodeficiency centromeric region instability and facial anomalies syndrome (ICF), Riddle syndrome, Hoyeraal–Hreidarsson syndrome (HHS), phosphoglucomutase 3 (PGM3) deficiency), and chronic granulomatous disease (CGD)—is based on the International Union of Immunological Societies: Update 2019 Primary Immunodeficiency Disease Committee Report on Inborn errors of Immunity [22,23].

Ataxia-telangiectasia-like disorder is a very rare disease due to mutations in the MRE11 gene, which is involved in DNA repair. The encoded Mre11 protein is biochemically linked to the ATM protein. It is a part, together with NBN and RAD50, of the trimeric protein complex MRN. The MRN complex has a direct role in DNA repair by both homologous recombination and non-homologous end-joining repair pathways, and it is also involved in the activation of ATM, and thus in the triggering of cell cycle checkpoints [24]. Patients present with a neurological picture, including involuntary movements and central and peripheral neuropathy, whose symptoms arise later and progress slower than AT. Moreover, they do not develop telangiectasias; the α-fetoprotein level is normal and immunological abnormalities are milder than those observed in AT [24].

Adenosine deaminase (ADA) deficiency is a severe CID due to an autosomal recessive mutation in the ADA gene, which encodes adenosine deaminase, an enzyme essential for the deamination of adenosine and deoxyadenosine in the purine salvage pathway [25]. Impaired enzyme function results in the accumulation of toxic metabolites, adenosine, deoxyadenosine, and deoxyadenosine triphosphate (dATP). dATP inhibits ribonucleotide reductase, a critical enzyme for DNA synthesis. The clinical phenotype of ADA patients is characterized by recurrent severe infections, skeletal abnormalities, hepatic and renal diseases, behavioral disorders, and autoimmune diseases [25,26]. Neurological manifestations include ataxia, motor dysfunction, hypotonia, nystagmus, development delay, cognitive impairment, reduced verbal expression, learning disability, hyperactivity, attention deficit, seizure, and sensorineural deafness [26]. The immunological findings include lymphopenia and the impairment of both cellular and humoral immunity due to abnormal T, B, and NK cell development and function. Ataxia, as the predominant sign of AT, is decisive in the differential diagnosis. The extra-neurological manifestations, especially skeletal abnormalities; brain MRI images revealing leukoencephalopathy; and the dilation of either ventricles or subarachnoidal spaces are of additional help in the differential diagnosis approach.

Purine nucleoside phosphorylase (PNP) deficiency is caused by an autosomal recessive mutation of the PNP gene, which encodes an enzyme playing an essential role in the purine salvage pathway [27]. The PNP mutations result in the accumulation of purine metabolites, which are toxic for lymphocytes [27]. PNP-deficient patients typically present with recurrent and unusual infections involving the respiratory and gastrointestinal systems; two thirds of patients suffer from neurologic abnormalities and one third from autoimmune disorders [28]. Neurological symptoms include ataxia, motor system dysfunction, cerebral palsy, hyper/hypotonia, spastic paresis, disequilibrium, development delay, cognitive impairment, and behavioral disorders [27,29]. Patients show lymphopenia with low CD3+, CD4+, and CD8+ lymphocyte subsets; immunoglobulin levels and B cell function may be affected to variable degrees [28]. Despite the clinical similarities, the elevation of α-fetoprotein and the finding of cerebellar atrophy orient towards the diagnosis of AT.

Nijmegen breakage syndrome is a rare, autosomal recessive disease resulting from a mutation in the nibrin (NBN) gene. The protein encoded (nibrin) is one of the three major components, together with MRE11 and RAD50, of the MRN complex. Patients present with microcephaly, cognitive development delay, behavioral or psychiatric disorders, and other clinical abnormalities described in detail in Table 1, but do not share ataxia and telangiectasias with AT. The immunodeficiency is more severe than in AT. Laboratory examinations show panhypogammaglobulinemia, profound T cell cytopenia, and a reduced T cell response to mitogens. Moreover, patients are at high risk of developing lymphoid malignancies [30].

DNA ligase IV deficiency is an autosomal recessive disease caused by hypomorphic mutations in the DNA ligase IV gene, encoding a key component of the non-homologous end-joining (NHEJ) pathway, the principal pathway employed to repair double-stranded DNA breakage. The affected patients present with microcephaly, ataxia, growth, and cognitive delays; the presence of facial dysmorphisms and bone deformities may help in the AT differential diagnosis [31]. Immune abnormalities may range from a clinical phenotype compatible with CID to a milder presentation with various degrees of lymphopenia and hypogammaglobulinemia, underlying the appearance of severe recurrent infections and an increased risk for lymphoid malignancies [32].

*Cernunnos/XLF deficiency* is a very rare autosomal recessive syndrome due to a mutation of the NHEJ1 gene (2q35), which interacts closely with LIG4 and has a similar clinical phenotype [33]. Patients present with profound microcephaly, developmental delay, facies dysmorphisms, and recurrent infections. Immune findings range from mild to severe B and T lymphopenia (NK cells are normal) and hypogammaglobulinemia (low IgG and IgA isotypes) with normal or increased IgM levels [33].

Riddle syndrome is a rare autosomal recessive syndrome due to mutations in the gene RNF168, encoding an E3 ubiquitin ligase protein involved in DNA double-strand break repair. Its clinical profile includes mild motor control and ataxia, conjunctival telangiectasis, and recurrent infections [34].

Hoyeraal–Hreidarsson syndrome is an X-linked syndrome caused by mutations in the DKC1 gene (Xq28), encoding the nucleolar protein dyskerin, that interacts with the human telomerase RNA complex. The neurologic manifestations include microcephaly ataxia, epilepsy, and delays in neurocognitive development. Hyperpigmentation, nail dystrophy, oral mucocutaneous lesions, and the early onset of bone marrow failure resulting in pancytopenia differentiate the syndrome from AT. The immunologic profile shows a progressive combined immunodeficiency [35].

Immunodeficiency centromeric region instability and facial anomalies syndrome is a rare autosomal recessive disease due to mutations in a DNA methyltransferase gene [36] and characterized by specific chromosomal rearrangements targeted adjacent to the centromeric region of chromosome 1 and/or 16. Patients suffer from neurologic manifestations including ataxia, hypotonia, and psychomotor delay [36]. The presence of dysmorphic facial features (see Table 1), macrocephaly, and, in many cases, of growth failure differentiates ICF from AT. Laboratory investigations show, in most cases, hypogammaglobulinemia with a normal B cell count; T cells are decreased in approximately half of the patients [37].

Common variable immunodeficiency disease (CVID) is an immune disorder characterized by low levels of protective antibodies and a wide spectrum of clinical manifestations due to immunodeficiency and immune dysregulation. Neurologic dysfunction is less frequently reported in CVID and its complex nature includes infectious, autoimmune/inflammatory, and vascular origins. CVID’s neurologic manifestations similar to AT are related to free-radical-mediated neuronal damage due to vitamin E deficiency and include ataxia, hyporeflexia, paresthesia, and rarely myoclonic dystonia [38].

Phosphoglucomutase 3 (PGM3) deficiency is an autosomal recessive form of hyper-IgE syndrome (HIES), due to hypomorphic PGM3 gene mutations, encoding for PGM3, which is involved in the protein glycosylation pathway, and it causes a multisystem phenotype. Patients present with early onset of neurological impairment, including ataxia, hypotonia (see Table 1), and the clinical triad of the HIES phenotype: recurrent pneumonia, recurrent skin abscesses, and highly elevated serum IgE [39].

Chronic granulomatous disease (CGD) is the most common inherited phagocytic cell defect, leading to impaired microbicidal function. It is caused by mutations of genes encoding the subunits of the nicotinamide adenine dinucleotide phosphate (NADPH) oxidase (PHOX) complex: gp91phox (X-linked form), p22phox, p47phox, and p67pxox (autosomal and recessive forms), whose deficiency results in a defect in superoxide production. The clinical picture is characterized by recurrent life-threatening bacterial and fungal infections and tissue granuloma formation, which rarely affects the central nervous system (CNS), resulting in CNS granulomatous disease [40].

## 4. Conclusions

Ataxia telangiectasia (AT) is a multisystemic neurodegenerative disease with progressive evolution. The onset time of the different manifestations and the rapidity of progression of the disease lead to phenotypic variability in its presentation in childhood. When immunodeficiency is the first feature, in the absence of the cardinal clinical neurological signs, the diagnosis is extremely challenging. An immunological profile that should raise suspicion is preferably the T(−) B(−) NK(+) CID phenotype with preserved NK cell count (NK cells do not undergo V(D)J recombination) and raised IgM. Neurological manifestations may appear later than recurrent infections, leading to misdiagnosis or delayed diagnosis. Therefore, the physical examination of a child with clinical manifestations of immunodeficiency, immune dysregulation, or malignancy needs to include an accurate neurologic evaluation. Similarly, strong suspicion of an underlying immune defect is relevant in any case of a clinical picture arising with a neurological pattern associated with or followed by severe, recurrent, or unusual infections. In patients presenting with features of immunodeficiency and neurological involvement, the laboratory routine must include, together with the immunological profile, AFP analysis. The diagnosis of radiosensitivity is difficult and available in only a few laboratories. Regarding this issue, we provide a review of the primitive inborn defects associated with neurological signs, to contribute to untangling the complex diagnostic procedure of the patient with AT, before the key signs of the disease are overall expressed.

## Figures and Tables

**Table 1:** Inborn errors of immunity with neurologic features: the differential diagnosis with ATP.

	Similarities in Clinical and Laboratory Features with AT	Differentiating Features Not Commonly Observed in AT
Ataxia-Telangiectasia-like Disorders (ATDL)	-Ataxia -Involuntary movements (tremor, chorea, dystonia) -Central/peripheral neuropathy	-Late onset -Less severe phenotype, slow progression -Telangiectasias, immunodeficiency, AFP increase rarely present
Adenosine Deaminase (ADA) Deficiency	-Ataxia -Motor dysfunction -Hypotonia -Combined immunodeficiency, recurrent severe infections	-Neurocognitive delay, epilepsy -Sensorineural deafness -Skeletal abnormalities -Normal AFP level -Brain MRI: leukoencephalopathy, dilatation of ventricles and subarachnoid spaces
Purine Nucleoside Phosphorylase (PNP) Deficiency	-Ataxia -Involuntary movements (tremor, chorea, dystonia) -Hyper/hypotonia -T cell lymphopenia, recurrent, unusual infections -Autoimmune disorders (one third of patients)	-Delay in neurocognitive development -Sensorineural deafness -Normal level of AFP -Brain MRI: absence of cerebellar atrophy
Nijmegen Breakage Syndrome (NBS)	-Severe combined immunodeficiency, severe infections -Radiosensitivity -Increased susceptibility to lymphoid malignancies	-Intrauterine growth retardation -Microcephaly -Facial dysmorphism (mid-facial prominence accentuated by the obliquity of the forehead and the receding jaw) -Neurocognitive delay, behavioral disorders -Skeletal abnormalities (clinodactyly of the fifth finger and partial syndactyly of the second and third toes)
DNA Ligase IV Deficiency (LIG4)	-Ataxia -Growth delay	-Microcephaly, facial dysmorphisms, bone deformations -Cognitive delay, learning difficulties -Skin abnormalities (psoriasis, eczema, erythroderma)
Cerunnos/XLF Deficiency	-Recurrent infections -Lymphopenia, hypogammaglobulinemia	-Microcephaly -Dysmorphic features, including “bird-like” facial dysmorphism
Riddle Syndrome	-Mild motor control -Ataxia -Conjunctival teleangectasias -Radiosensitivity and cancer susceptibility -Combined immunodeficiency -Recurrent infections -Increase in AFP	-Psychomotor retardation -Atopy, serum IgE elevation -Sensorineural hearing loss
Hoyeraal–Hreidarsson Syndrome (HHS)	-Ataxia -Hypotonia -Progressive combined immunodeficiency	-Prenatal growth retardation -Microcephaly -Neurocognitive delay -Epilepsy -Pancytopenia -Hyperpigmentation, nail dystrophy -Premalignant leukoplakia oral and gastrointestinal
Immunodeficiency, Centromeric Region Instability and Facial Anomalies Syndrome (ICF)	-Ataxia -Hypotonia -Hypo/agammaglobulinemia, decrease in T cell count (half of cases)	-Psychomotor delay -Facial abnormalities: hypertelorism and epicant folds, micrognathia, low ear implantation, and macroglossia -Macrocephaly
Common Variable Immunodeficiency Disease (CVID)	-Humoral immunodeficiency, recurrent infections -Ataxia, dysarthria, tremor -Paraesthesia -Myoclonic dystonia	-Entheropathy -Vitamin E deficiency -Guillain Barré Syndrome
Phosphoglucomutase 3 (PGM3) Deficiency	-Ataxia, hypotonia, dysarthria -Thymic dysfunction, recurrent infections -Cancer susceptibility	-Retinitis pigmentosa
Chronic Granulomatous Disease (CGD)	-CNS granulomatous disease -Cutaneous granulomas, chronic inflammation -Recurrent severe infections	-Defective bactericidal function -Hypergammaglobulinemia -Normal level of AFP

## References

[1] Rothblum-Oviatt C., Wright J., Lefton-Greif M.A., McGrath-Morrow S.A., Crawford T.O., Lederman H.M. (2016). Ataxia telangiectasia: A review. Orphanet J. Rare Dis..

[2] Savitsky K., Bar-Shira A., Gilad S., Rotman G., Ziv Y., Vanagaite L., Tagle D.A., Smith S., Uziel T., Sfez S. (1995). A single ataxia telangiectasia gene with a product similar to PI-3 kinase. Science.

[3] Bredemeyer A.L., Sharma G.G., Huang C.-Y., Helmink B.A., Walker L.M., Khor K.C., Nuskey B., Sullivan K.E., Pandita T.K., Bassing C.H. (2006). ATM stabilizes DNA double-strand-break complexes during V(D)J recombination. Nature.

[4] Alyasin S., Esmaeilzadeh H., Ebrahimi N., Nabavizadeh S.H., Nemati H. (2019). Clinical Presentation of Ataxia-Telangiectasia. Arch. Iran. Med..

[5] van Os N.J., Chessa L., Weemaes C.M., van Deuren M., Fiévet A., van Gaalen J., Mahlaoui N., Roeleveld N., Schrader C., Schindler D. (2019). Genotype-phenotype correlations in ataxia telangiectasia patients with ATM c.3576G>A and c.8147T>C mutations. J. Med. Genet..

[6] Sahama I., Sinclair K., Pannek K., Lavin M., Rose S. (2014). Radiological imaging in ataxia telangiectasia: A review. Cerebellum.

[7] Cabana M.D., Crawford T.O., Winkelstein J.A., Christensen J.R., Lederman H.M. (1998). Consequences of the delayed diagnosis of ataxia-telangiectasia. Pediatrics.

[8] Bredemeyer A.L., Helmink B.A., Innes C.L., Calderon B., McGinnis L.M., Mahowald G.K., Gapud E.J., Walker L.M., Collins J.B., Weaver B.K. (2008). DNA double-strand breaks activate a multi-functional genetic program in developing lymphocytes. Nature.

[9] Nowak-Wegrzyn A., Crawford T.O., Winkelstein J.A., Carson K.A., Lederman H.M. (2004). Immunodeficiency and infections in ataxia-telangiectasia. J. Pediatr..

[10] Boyarchuk O., Makukh H., Kostyuchenko L., Yarema N., Haiboniuk I., Kravets V., Shulhai O., Tretyak B. (2021). TREC/KREC levels in children with ataxia-telangiectasia. Immunol. Res..

[11] McGrath-Morrow S.A., Collaco J.M., Detrick B., Lederman H.M. (2016). Serum Interleukin-6 Levels and Pulmonary Function in Ataxia-Telangiectasia. J. Pediatr..

[12] Paller A.S., Massey R.B., Curtis M.A., Pelachyk J.M., Dombrowski H.C., Leickly F.E., Swift M. (1991). Cutaneous granulomatous lesions in patients with ataxia-telangiectasia. J. Pediatr..

[13] Reiman A., Srinivasan V., Barone G., Last J.I., Wootton L.L., Davies E.G., Verhagen M.M., Willemsen M.A., Weemaes C.M., Byrd P.J. (2011). Lymphoid tumours and breast cancer in ataxia telangiectasia; substantial protective effect of residual ATM kinase activity against childhood tumours. Br. J. Cancer.

[14] van Os N.J., Haaxma C.A., van der Flier M., Merkus P.J., van Deuren M., de Groot I.J., Loeffen J., van de Warrenburg B.P., Willemsen M.A., A-T Study Group (2017). Ataxia-telangiectasia: Recommendations for multidisciplinary treatment. Dev. Med. Child. Neurol..

[15] Lavin M.F., Gueven N., Bottle S., Gatti R.A. (2007). Current and potential therapeutic strategies for the treatment of ataxia-telangiectasia. Br. Med. Bull..

[16] Biagiotti S., Bianchi M., Rossi L., Chessa L., Magnani M. (2019). Activation of NRF2 by dexamethasone in ataxia telangiectasia cells involves KEAP1 inhibition but not the inhibition of p38. PLoS ONE.

[17] Buoni S., Zannolli R., Sorrentino L., Fois A. (2006). Betamethasone and improvement symptoms in ataxia-telangiectasia. Arch. Neurol..

[18] Martire B., Azzari C., Badolato R., Canessa C., Cirillo E., Gallo V., Graziani S., Lorenzini T., Milito C., Panza R. (2018). Vaccination in immunocompromised host: Recommendations of Italian Primary Immunodeficiency Network Centers (IPINET). Vaccine.

[19] Herskind A., Greisen G., Nielsen J.B. (2014). Early identification and intervention in cerebral palsy. Dev. Med. Child. Neurol..

[20] Salman M.S., Ikeda K.M. (2013). The syndrome of infantile-onset saccade initiation delay. Can. J. Neurol. Sci./J. Can. des Sci. Neurol..

[21] Anheim M., Tranchant C., Koenig M. (2012). The autosomal recessive cerebellar ataxias. N. Engl. J. Med..

[22] ESID Registry—Working Definitions for Clinical Diagnosis of PID 2019. https://esid.org/Working-Parties/Registry-Working-Party/Diagnosis-criteria.

[23] Dehkordy S.F., Aghamohammadi A., Ochs H.D., Rezaei N. (2011). Primary immunodeficiency diseases associated with neurologic manifestations. J. Clin. Immunol..

[24] Taylor A.M., Groom A., Byrd P.J. (2004). Ataxia-telangiectasia-like disorder (ATLD)—Its clinical presentation and molecular basis. DNA Repair..

[25] Sauer A.V., Aiuti A. (2009). New insights into the pathogenesis of adenosine deaminase-severe combined immunodeficiency and progress in gene therapy. Curr. Opin. Allergy Clin. Immunol..

[26] Nofech-Mozes Y., Blaser S.I., Kobayashi J., Grunebaum E., Roifman C.M. (2007). Neurologic abnormalities in patients with adenosine deaminase deficiency. Pediatr. Neurol..

[27] Cohen A., Grunebaum E., Arpaia E., Roifman C.M. (2000). Immunodeficiency caused by purine nucleotide phosphorylase deficiency. Immunol. Allergy Clin. N. Am..

[28] Dalal I., Grunebaum E., Cohen A., Roifman C. (2001). Two novel mutations in a purine nucleoside phosphorylase (PNP)-deficient patient. Clin. Genet..

[29] Tabarki B., Yacoub M., Tlili K., Trabelsi A., Dogui M., Essoussi A.S. (2003). Familial spastic paraplegia as the presenting manifestation in patients with purine nucleoside phosphorylase deficiency. J. Child. Neurol..

[30] Digweed M., Sperling K. (2004). Nijmegen breakage syndrome: Clinical manifestation of defective response to DNA double-strand breaks. DNA Repair..

[31] Chistiakov D.A., Voronova N.V., Chistiakov A.P. (2009). Ligase IV syndrome. Eur. J. Med. Genet..

[32] Enders A., Fisch P., Schwarz K., Duffner U., Pannicke U., Nikolopoulos E., Peters A., Orlowska-Volk M., Schindler D., Friedrich W. (2006). A severe form of human combined immunodeficiency due to mutations in DNA ligase IV. J. Immunol..

[33] Buck D., Malivert L., de Chasseval R., Barraud A., Fondanèche M.-C., Sanal O., Plebani A., Stéphan J.-L., Hufnagel M., le Deist F. (2006). Cernunnos, a novel nonhomologous end-joining factor, is mutated in human immunodeficiency with microcephaly. Cell.

[34] Stewart G.S., Panier S., Townsend K., Al-Hakim A.K., Kolas N.K., Miller E.S., Nakada S., Ylanko J., Olivarius S., Mendez M. (2009). The RIDDLE syndrome protein mediates a ubiquitin-dependent signaling cascade at sites of DNA damage. Cell.

[35] Yang C.R., Wei Q., Jiang M., Zhang X.B., Zhang Z.X., Nong G.M. (2022). Hoyeraal-Hreidarsson syndrome with combined immunodeficiency and enterocolitis caused by a DCK1 gene variant. Chin. J. Pediatr..

[36] Hagleitner M.M., Lankester A., Maraschio P., Hulten M., Fryns J.P., Schuetz C., Gimelli G., Davies E.G., Gennery A., Belohradsky B.H. (2007). Clinical spectrum of immunodeficiency, centromeric instability and facial dysmorphism (ICF syndrome). J. Med. Genet..

[37] Ehrlich M. (2003). The ICF syndrome, a DNA methyltransferase 3B deficiency and immunodeficiency disease. Clin. Immunol..

[38] Nguyen J.T.U., Green A., Wilson M.R., DeRisi J.L., Gundling K. (2016). Neurologic Complications of Common Variable Immunodeficiency. J. Clin. Immunol..

[39] Sassi A., Lazaroski S., Wu G., Haslam S.M., Fliegauf M., Mellouli F., Patiroglu T., Unal E., Ozdemir M.A., Jouhadi Z. (2014). Hypomorphic homozygous mutations in phosphoglucomutase 3 (PGM3) impair immunity and increase serum IgE levels. J. Allergy Clin. Immunol..

[40] Dasovic B., Borys E., Schneck M.J. (2022). Granulomatous Diseases of the Central Nervous System. Curr. Neurol. Neurosci. Rep..

